# Inhibition of ACE2–S Protein Interaction by a Short Functional Peptide with a Boomerang Structure

**DOI:** 10.3390/molecules29133022

**Published:** 2024-06-26

**Authors:** Yuping Wei, Ziyang Liu, Man Zhang, Xingyan Zhu, Qiuhong Niu

**Affiliations:** 1 School of Life Science, Nanyang Normal University, Nanyang 473061, China; fuziannwei@163.com (Y.W.); 18538992007@163.com (Z.L.); 18313948872@163.com (X.Z.); 2 Research Center of Henan Provincial Agricultural Biomass Resource Engineering and Technology, Nanyang Normal University, Nanyang 473061, China; 18737779319@163.com; 3 State Key Laboratory of Biochemical Engineering, Institute of Process Engineering, Chinese Academy of Sciences, Beijing 100190, China; 4 Department of Oncology, Nanyang First People’s Hospital, Nanyang 473002, China

**Keywords:** SARS-CoV-2, ACE2, molecular simulation, short functional peptide, proline boomerang structure

## Abstract

Considering the high evolutionary rate and great harmfulness of severe acute respiratory syndrome coronavirus 2 (SARS-CoV-2), it is imperative to develop new pharmacological antagonists. Human angiotensin-converting enzyme-2 (ACE2) functions as a primary receptor for the spike protein (S protein) of SARS-CoV-2. Thus, a novel functional peptide, KYPAY (K5), with a boomerang structure, was developed to inhibit the interaction between ACE2 and the S protein by attaching to the ACE2 ligand-binding domain (LBD). The inhibition property of K5 was evaluated via molecular simulations, cell experiments, and adsorption kinetics analysis. The molecular simulations showed that K5 had a high affinity for ACE2 but a low affinity for the cell membrane. The umbrella sampling (US) simulations revealed a significant enhancement in the binding potential of this functional peptide to ACE2. The fluorescence microscopy and cytotoxicity experiments showed that K5 effectively prevented the interaction between ACE2 and the S protein without causing any noticeable harm to cells. Further flow cytometry research indicated that K5 successfully hindered the interaction between ACE2 and the S protein, resulting in 78% inhibition at a concentration of 100 μM. This work offers an innovative perspective on the development of functional peptides for the prevention and therapy of SARS-CoV-2.

## 1. Introduction

The global COVID-19 pandemic, caused by the pathogen known as severe acute respiratory syndrome coronavirus 2 (SARS-CoV-2), a positive-sense single-stranded RNA virus, has affected a total of approximately 559.47 million humans, with around 6.36 million fatalities [1]. Presently, although the SARS-CoV-2 vaccine provides excellent protection against SARS-CoV-2 infection, vaccinated individuals may exhibit increased vulnerability to highly pathogenic viruses, especially under conditions of high exposure, and the ongoing mutations of the virus also present challenges to the effectiveness of current vaccinations [2,3]. A proportion of patients who recover from SARS-CoV-2 will have persistent or new symptoms [4], and infection with novel coronaviruses in patients with underlying disease results in higher mortality rates [5]. Thus, the development of antiviral drugs remains a top priority.

Despite a high evolutionary rate leading to the continuous emergence of novel lineages, there is evidence that the mutations share the same cell entrance receptor, known as angiotensin-converting enzyme II (ACE2) [6,7,8]. Usually, coronaviruses employ the homotrimer spike glycoprotein (S protein) located in their envelope to bind to their cellular receptors. This specific binding of the SARS-CoV-2 spike (S) protein to the ACE2 protein marks the initial step in the virus’s invasion into human cells [9]. The S protein is typically cleaved into the spike 1 and spike 2 subunits (S1 and S2 proteins) by furin proteases; the S1 subunit is responsible for the specific binding to ACE2, while the S2 subunit facilitates invasion into cells [10,11,12]. Previous research successfully determined the crystal structure of the SARS-CoV-2 receptor-binding domain (RBD) in complex with human ACE [13,14]; the detailed structure of the SARS-CoV-2 S1 protein RBD bound to ACE2 was resolved, revealing that region 433–472 directly interacts with human ACE2, suggesting its potential importance to cell transduction [15,16]. Therefore, inhibiting the specific ligand–receptor interaction between the SARS-CoV-2 S1 protein and ACE2 may prevent the virus from invading human cells [17,18]. Currently, the research and development of therapeutic drugs for COVID-19 have continuously garnered worldwide interest [19], with a specific focus on the development of inhibitors that block viral cell infection. The efficacy of recombinant ACE2 in capturing the SARS-CoV-2 S protein has been validated; however, ACE2 is not optimal for intranasal delivery [20,21,22], due to its large size and limited density of binding sites. Instead, peptide inhibitors derived from the S1 protein that have a high affinity for ACE2 are promising candidates [23,24].

Due to the rapid development of algorithms and hardware over the past two decades, molecular simulation has become a reliable method for researching biomolecular systems and designing novel drugs [25]. Compared to previous experiments, in this study, a revolutionary strategy was introduced into the rational design of ACE2–S protein binding inhibitors. The interactions and binding sites between ACE2 and the S protein were initially examined to further identify the critical ligand residues, surface characteristics, and topographical features of the receptor. Then, a novel short functional peptide with a boomerang structure, based on the key residues of the S protein RBD and targeting the surface properties and topography of the ACE2 LBD, was developed by molecular simulation. A potential peptide library was created by considering the interaction sites and important residues, and subsequently, candidate peptides were obtained, screened, and confirmed by molecular simulations. Following this, cellular assays were carried out to verify and evaluate the peptides’ capacity to inhibit the interaction between the S1 protein and the ACE2 receptor. Further biophysical studies of K5 were performed in vitro.

## 2. Results

### 2.1. Analysis of ACE2–S Protein Interaction and Functional Peptide Design

ACE2 functions as the primary receptor for SARS-CoV-2 within the human body, providing the affinity between the viral S protein RBD and the host cells during the early stage of SARS-CoV-2 infection [26,27]. Hence, a novel rational design strategy, based on the key residues of the S protein and adapted to the surface properties and topography of the ACE2 LBD, was employed to develop short functional peptides that inhibit the interaction between ACE2 and the S protein, directly preventing and suppressing viral infection.

**Figure 1:**
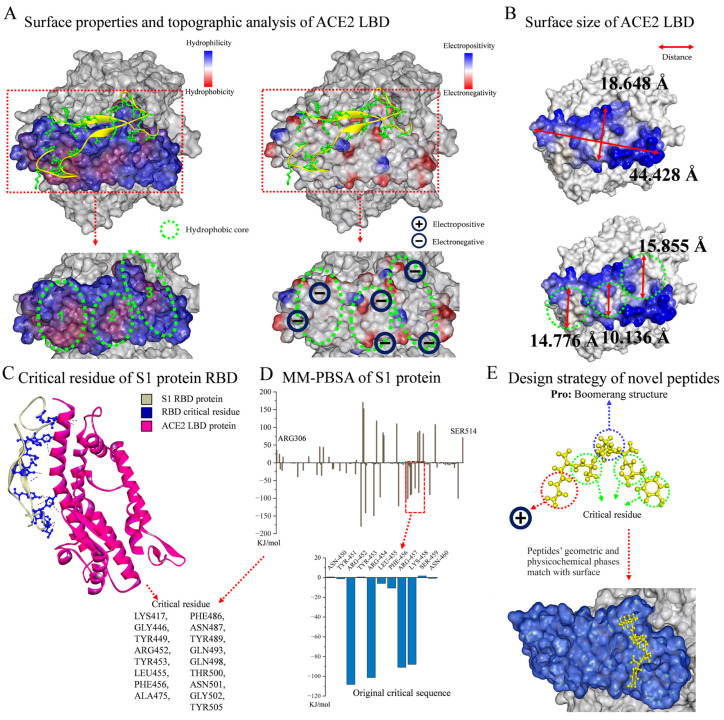
Analysis of ACE2–S protein interaction and functional peptide design. (**A**) Surface properties and topographic analysis of ACE2 LBD. (**B**) Surface size of ACE2 LBD. (**C**) DSV analysis of critical residue of S1 protein RBD. (**D**) Residue contribution analysis of S1 protein by MM-PBSA method. (**E**) Design strategy of novel peptides.

As shown in Figure 1A, the surface properties and topographic analysis of the ACE2 LBD revealed that it contained SER19-LEU85, LEU351-ILE358, which was a mountainous protrusion with electronegative edges (approx. 18.648 Å × 44.428 Å, Figure 1B). It is worth noting that the ACE2 LBD has three concave hydrophobic cores (approx. diameter: 13.589 Å, Figure 1B), and the outer edges of these cores display significant electronegativity, giving this structure a very distinctive nature. The surface topography of the ACE2 LBD was viewed from different angles, as shown in Figure S1. In accordance with previous reports [28,29] and by evaluating the residue interaction network and the contribution of each residue in the S protein RBD, several crucial residues which engaged in non-covalent interactions and had a strong affinity were further identified, including Lys417, Gly446, Tyr449, Arg452, Tyr453, Leu455, Phe456, Ala475, Phe486, Asn487, Tyr489, Gln493, Gln498, Thr500, Asn501, Gly502, and Tyr505 (Figure 1C). Hence, a revolutionary rational design strategy was introduced for ACE2–S protein binding inhibitors. Figure 1E illustrates the design strategy for novel peptides, guided by the following principles: First was the boomerang structure formed by proline. The central part of the functional peptide was proline, and its unique structure resulted in a boomerang-like shape for the whole peptide, facilitating the geometric compatibility of ligands to receptors by allowing peptides to easily fit into the concave or attach to the protrusion in the S protein RBD region. Second was the development of functional peptides via key residues. The residues of functional peptides were selected from key residues to ensure their affinity for receptors. For the N-terminus, electropositive residues were selected to interact with the electronegative edges of the LBD or hydrophobic cores. Third were short peptides. Short peptides are non-toxic, non-immunogenic, and easy to synthesize. The hydrophobic core has a diameter of around 15 Å, and the breadth of the LBD is roughly 18 Å, which are an appropriate size for five residues; thus, a peptide library containing 2000 pentapeptides was established. The original critical sequence, YRLFR (Y5), of the S protein (Figure 1D) was selected as the control group for new functional peptide design. 

### 2.2. Molecular Simulation

**Figure 2:**
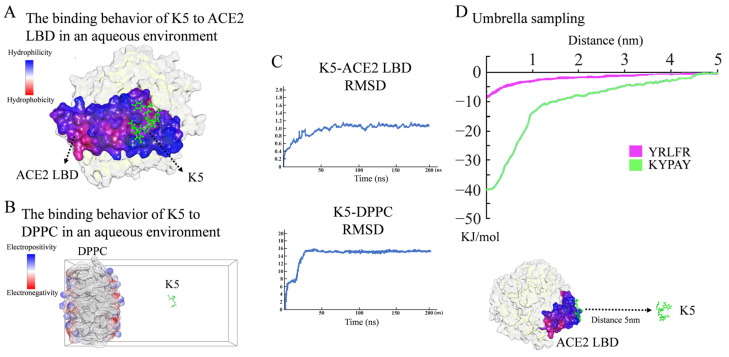
Molecular simulation analysis of peptide. (**A**) Binding behavior of K5 to ACE2 LBD in aqueous environment. Snapshots of K5-ACE2 LBD system after 200 ns simulation. (**B**) Interaction between K5 and DPPC bilayer membrane in aqueous environment. Snapshots of K5-DPPC system after 200 ns simulation. (**C**) RMSD values of K5-ACE2 LBD system and K5-DPPC system. (**D**) US simulation and ΔG values of K5 and Y5 bound to ACE2 LBD.

The preliminary screening of functional peptides was carried out by molecular docking, while MD simulations were used for further validation and screening. In accordance with the free binding energy, RMSD, and geometry criteria described in previous studies [30,31], the binding free energy (E_binding_) between a peptide and ACE2 was required to be lower than −4.3 kcal/mol, and only peptides with a high affinity, stable conformation, and desirable binding sites with the ACE2 LBD were chosen for further study. As demonstrated in Table S1, 14 sequences with high E_binding_ were selected. Further molecular dynamics (MD) simulations revealed that, among these 14 sequences, only KYPAY (K5) could spontaneously bind to the ACE2 LBD (Figure 2A), and the RMSD indicated that all systems quickly reached equilibrium (Figure 2C). Further research revealed that the E_binding_ of K5 to the ACE2 LBD was -6.91 Kcal/mol, which was about twice as high as that of the control sequence Y5, suggesting that the affinity of K5 was significantly increased. The E_binding_ of K5 to a DPPC membrane was −3.17 Kcal/mol, suggesting that K5 has low affinity to membranes. The MD simulation results showed that the binding site of K5 was located at concave hydrophobic core 3. Proline was oriented towards the concave center of the hydrophobic core, whereas the residues on both sides attached to the concave area. The positively charged N-terminal bonded to the negatively charged edge, which was consistent with the initial design. Meanwhile, the MD simulation of the K5-DPPC system demonstrated that K5 was not bound to the cell membrane after a 200 ns simulation, confirming its enhanced selectivity (Figure 2B). Notably, the MD simulations showed that the functional peptide did not form any secondary structures, indicating that the secondary structure of K5 might not be directly related to its function. The fluctuation in the RMSD revealed that all systems quickly obtained equilibrium (Figure 2C).

To further validate the affinity of K5, the binding potential (ΔG) of the peptide–ACE2 system was calculated using US simulations and further compared with a core sequence of the S1 protein (Y5, YRLFR). As illustrated in Figure 2D, the ΔG values of the K5-ACE2 systems were −40 kJ/mol, while that of the Y5-ACE2 system was −8 kJ/mol. The US simulation results revealed that the functional peptide increased the binding potential, implying that K5 has a high potential to interact with ACE2. The binding energy of our designed functional peptide to the ACE2 protein is 5 times that of the original key sequence. Thus, K5 was selected and synthetized (Figures S2–S4) as the functional peptide candidate for the cytotoxicity and cellular adsorption tests. 

### 2.3. Cytotoxicity of K5

**Figure 3:**
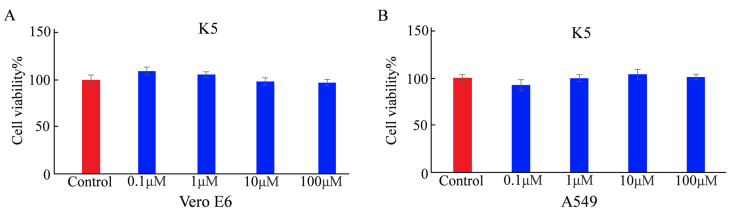
Cytotoxicity of K5 analyzed by MTT assay. (**A**) Cell viability of Vero E6 cells incubated with K5 at concentrations of 0.1, 1, 10, and 100 μM for 24 h. (**B**) Cell viability of A549 cells cultured with K5 at concentrations of 0.1, 1, 10, and 100 μM for 24 h.

The cytotoxicity of a functional peptide is a crucial factor for applications involving biosystems. Before determining the cellular adsorption behavior of K5, its cytotoxicity was determined using the MTT assay [32]. Vero E6 and A549 cells were each incubated for 24 h (h). The cell viability rates of Vero E6 were 108%, 105%, 96%, and 94% with a series of the functional peptide at different concentrations (0.1, 1, 10, and 100 μM, respectively). The cell viability rates of A549 cells were 93%, 100%, 103%, and 101% with a series of K5 at different concentrations (0.1, 1, 10, and 100 μM, respectively). There was no discernible change in cell viability under any of the tested conditions, indicating that K5 was not toxic at physiological pH (Figure 3).

### 2.4. Cellular Adsorption Behaviors of K5

**Figure 4:**
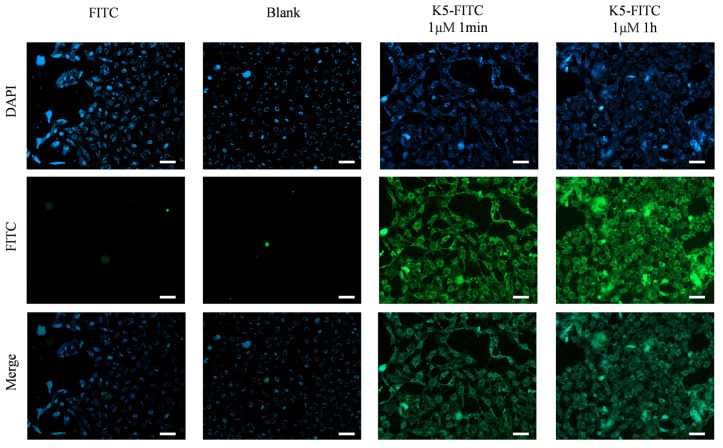
Cellular adsorption behaviors of K5. Fluorescence micrographs of cells incubated with 1 μM K5-FITC for 1 min and 1 h. Scale bar (white bar) = 50 μm.

The evaluation of the effectiveness of a receptor antagonist relies heavily on its target affinity [33]. Remarkably, as depicted in Figure 4, an obvious green fluorescence was detected in the presence of 1 μM of K5-FITC after only 1 min, and it became much stronger after 1 h, suggesting a very high cellular adsorption rate of the functional peptide. In contrast, untreated cells (Blank) and cells treated solely with FITC (FITC) did not exhibit any fluorescence, indicating that K5 has the ability to effectively adsorb onto cells.

### 2.5. Inhibition of ACE2–S Protein Interaction by K5

**Figure 5:**
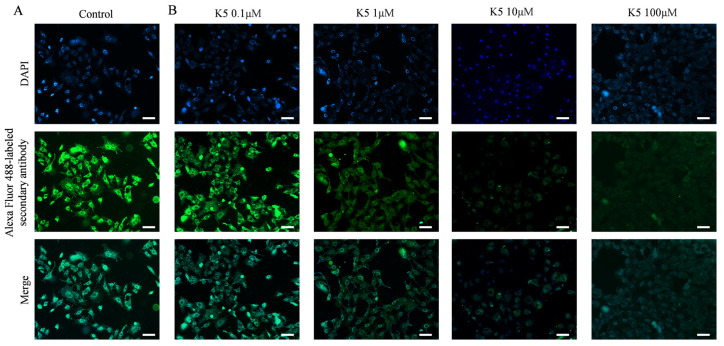
Blockage of S1 protein–ACE2 interaction by K5. (**A**) Visualization of S1 protein binding to ACE2 on cell surface. (**B**) Visualization of inhibition effect of S1 protein–ACE2 interaction by 0.1, 1, 10, and 100 μM K5. Scale bar (white bar) = 50 μm.

In order to investigate the ability of the functional peptide to inhibit S protein–ACE2 interaction, Vero E6 cells and A549 cells were subjected to pre-incubation with various concentrations of K5. Subsequently, the S1 protein was introduced for further incubation. The S1 proteins that specifically bound to ACE2 were further stained by using primary and secondary antibodies, resulting in bright green fluorescence on the cell surface. However, the binding of K5 to the ACE2 LBD could block the binding of the S1 protein to ACE2 and lead to a decrease in fluorescence. Compared to the control group (Figure 5A), Figure 5B illustrates a significant decrease in fluorescence in the presence of 1 μM of functional peptide, suggesting that the functional peptide performed effectively at micromolar concentrations. In addition, there was an impressive reduction in the fluorescence of Vero E6 cells as the concentration of functional peptide increased, and the fluorescence was almost completely quenched at a peptide concentration of 100 μM, implying that K5 effectively inhibited the binding of the S protein to ACE2. Clearly, as the binding of K5 to ACE2 increased, the binding of the S1 proteins to ACE2 was increasingly blocked, resulting in a fluorescence decrease with increasing K5 concentration. Similar performance was seen with the A549 cells (Figure S5).

**Figure 6:**
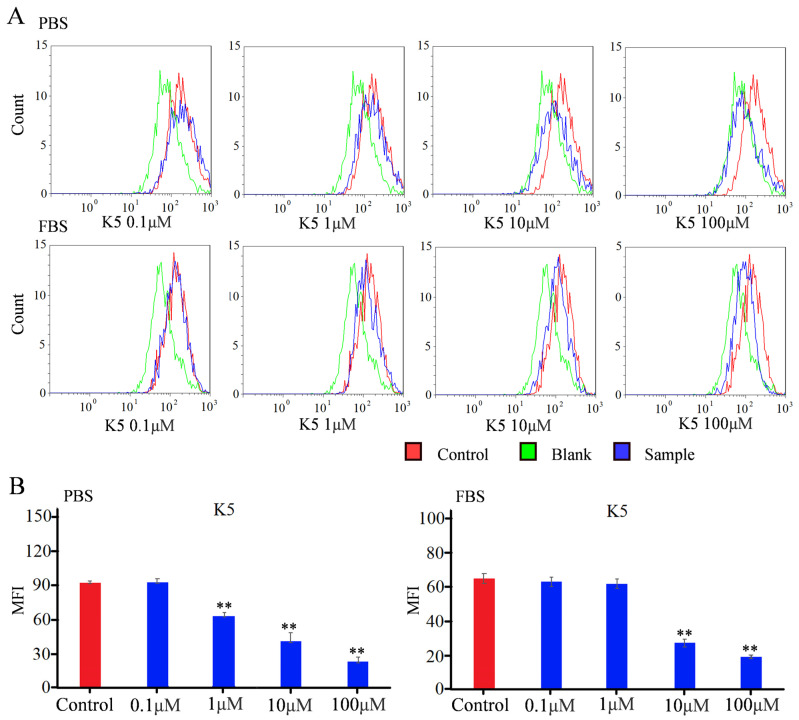
Flow cytometry analysis of inhibitory effect of K5 on S1 protein–ACE2 interaction. (**A**) FACS analysis of inhibitory effect of K5 on S1 protein–ACE2 interaction in PBS and FBS solution. (**B**) MFI analysis of inhibitory effect of K5 on S1 protein–ACE2 interaction in PBS and FBS solution. “**” represents a significant difference.

In order to detect the blocking effect of K5, a flow cytometry study was used to quantify the fluorescence intensity [34,35]. For the Vero E6 group in PBS conditions, the MFI did not exhibit a statistically significant change in the presence of 0.1 μM of functional peptide (Figure 6). However, the MFI decreased by approximately 25% in the presence of 1 μM of functional peptide, indicating that the functionality of the peptide was concentration-dependent, with a minimal effective concentration. The MFI exhibited a dramatic decline of 78% when incubated with 100 μM of K5. The value of the IC50 was calculated to be −6.7 μM, indicating that K5 had a remarkable blocking effect (Figure 6). For the A549 group in PBS conditions, the MFI decreased with increasing concentration of the functional peptide, with a significant reduction of 90% in the MFI with 100 μM of K5 (Figure S6). 

To more precisely mimic the conditions of the in vivo environment, the Vero E6 cells were further incubated with FBS containing K5 and the S1 protein in sequence. The flow cytometry data are shown in Figure 6. Although the K5 exhibited negligible efficacy at a concentration of 1 μM, there was still a notable decline in the MFI as the concentration of functional peptides increased. Furthermore, a decrease of 70% in the MFI was observed with 100 μM of K5, implying that K5 retained a robust inhibitory capacity within a plasma environment.

### 2.6. Biophysical Studies of K5

The MD simulation indicated that the functional peptide did not form any secondary structure, and further circular dichroism (CD) analysis showed that K5 exhibited random curling, implying that the secondary structure of the functional peptide is not intrinsically related to its function (Figure S7).

For a typical-affinity ligand, it is required for the K_d_ to be within the range of 10^−3^ to 10^−9^ M, preferably within 10^−5^ to 10^−7^ M [36]. The K_d_ can be determined by evaluating the adsorption kinetics. The adsorption curve of the functional peptides is shown in Figure S8, and the corresponding adsorption parameters were determined. The K_d_ of K5 was 1.25 × 10^−5^ M, suggesting that the functional peptide exhibits good affinity as a ligand.

## 3. Discussion

Proteins can bind to their receptors with high affinity when acting as ligands. However, the majority of protein inhibitors are focused on functional subunits rather than the entire protein, with only a few residues of the RBD sequence interacting with the receptor [37]. Hence, it would be feasible to use functional peptides as a mimic for protein ligands.

To date, only a small number of drug design studies have focused on the SARS-CoV-2 receptor, with a particular emphasis on ACE2 antagonists. Exogenous proteins have disadvantages such as low efficiency in drug delivery and immunogenicity, whereas small-molecule ligands pose limitations such as challenges for manufacturing and increased toxicity. Thus, direct interception of the amino acids of ACE2 could be a simple and effective method [38,39,40]. The length of the ACE2-derived peptide (CSNP1) is 16 amino acids [41]. The IC50 of the 3cvl-2 peptide with 16 amino acids is 4.90 μM [42]. The Covid extended_1 and Covid3 peptides, with IC50 values of 4–8 μM, contain 25 or more residues [43]. However, long peptides have secondary structures, could have immunogenicity, and could present problems with solid-phase production. Compared to these inhibitors, short functional peptides can be easily synthesized in large quantities, are low in cost, and do not cause an immune response when they consist of fewer than nine peptides [44]. Moreover, most short peptides lack a secondary structure, which do not have difficulty in refolding. Unfortunately, a reduction in the number of residues leads to a significant decline in the affinity of functional peptides to receptors. It was reported that the IC50 of short peptide inhibitor SAPs (EDLFYQ) was at the mM level, which is much higher than that of long peptide inhibitors. Thus, a smart peptide design strategy is crucial for short functional peptide design.

Generally, protein–protein interactions should be distinguished from peptide–protein interactions. The effective overall affinity of the whole protein ligand, such as its charge, hydrophobicity, and spatial structure, can compensate for the lack of affinity of individual residues of the RBD sequence [45], which is not present in peptide–protein binding. Simply interpreting the core sequence of a protein ligand as a peptide ligand will inevitably result in the loss of the overall affinity of the entire protein. Unlike in previous rational designs, in this study, peptide design encompassed more than just residue preferences and high-throughput screening. It emphasized the adaption of geometric and physicochemical features depending on the surface characteristics and topographical features of the receptor. For the first time, a short functional peptide with a boomerang structure was designed to match the surface properties and topography of the ACE2 LBD. Thanks to this innovative design strategy, the IC 50 of K5 was 6.7 μM; this pentapeptide inhibitor exhibited performance on par with that of longer peptide inhibitors from previous research while avoiding concerns such as toxicity, immunogenicity, and manufacturing challenges, making K5 an ideal potential drug.

It was noticed that the serum environment serves as the primary operational environment for the majority of biological inhibitors. In comparison to PBS, FBS exhibits a greater abundance of proteins. Consequently, FBS has the potential to cause stronger interference through greater non-specific adsorption, which is probably one of the reasons for the decrease in functional peptide efficiency in FBS. Inhibition of the S protein–ACE2 interaction remained effective as the concentration of functional peptides increased (a 50% reduction in the MFI in the presence of 10 μM of peptide). K5 maintained its effectiveness in the serum environment and exhibited outstanding ligand performance, indicating the feasibility of the newly established functional peptide design method.

## 4. Materials and Methods

### 4.1. Materials and Reagents

The Vero E6 cell line (monkey kidney cells) was acquired from Shanghai JinYuan Biotechnology Co., Ltd. (Shanghai, China). The A549 cell line (human cervical cancer cells) was acquired from the Cell Bank of the Typical Culture Preservation Committee of the Chinese Academy of Sciences (Shanghai, China). Peptide synthesis reagent *N*,*N*-Dimethylformamide (DMF), *O*-(Benzotriazol-1-yl)-*N*,*N*,*N*′,*N*′-tetramethyluronium tetrafluoro-borate (TBTU), 1-Hydroxybenzotriazole (HOBt), trifluoroacetic acid (TFA), *N*,*N*-Diisopropylethylamine (DIEA), 1,2-eth-anedithiol (EDT), thioanisole, isopropanol, phenol, piperidine, polypeptide (K5), and fluorescein isothiocyanate (FITC)-labeled polypeptide (K5-FITC) were purchased from GL Biochemistry (Shanghai, China). The SARS-CoV-2 Spike S1-His recombinant protein (S1 protein), human ACE2 protein, and SARS-CoV-2 Spike S1 antibody (rabbit primary antibody) were purchased from Sino Biological (Beijing, China). NHS-activated Sepharose 4 Fast Flow beads were purchased from Hushi Medicine Technology (Shanghai, China). A goat anti-rabbit secondary antibody (Alexa Fluor 488, Invitrogen) was purchased from Thermo Fisher Scientific (Beijing, China). Dulbecco’s modified Eagle’s medium (DMEM), trypsin, fetal bovine serum (FBS), and phosphate-buffered solution (PBS, pH 7.4) were obtained from Hyclone (Logan, UT, USA). Dimethyl sulfoxide (DMSO) was purchased from Aladdin (Shanghai, China). 5-Diphenyl-2-*H*-tetrazolium bromide (MTT), paraformaldehyde (PFA), 4′,6-diamidino-2-phenylindole (DAPI), glycine (Gly), and heparin were obtained from Solarbio (Beijing, China). 

### 4.2. Molecular Simulation

The crystal structure file of the SARS-CoV-2 S1 protein–ACE2 complex (ID: 7V8B) was obtained from the Protein Data Bank (https://www.rcsb.org/, accessed on 9 May 2024). The dipalmitoyl-phosphatidylcholine (DPPC) bilayer membrane model was downloaded from Gromacs (https://www.gromacs.org/, accessed on 13 May 2024). The 3D coordinates of functional peptides were generated by Discovery Studio Visualizer (DSV) 4.5. The surface properties and topography of the ACE2 LBD were investigated by DSV. The interactions between the ligand and receptor were further analyzed using the Poisson–Boltzmann surface area (MM-PBSA) method [46,47] and DSV [48,49]. The molecular docking was carried out by the AutoDock 4.2 package [50,51], and all settings were default. The molecular dynamics (MD) and umbrella sampling (US) simulations of the ligand–receptor systems were conducted for 200 ns within GROMACS 2019 software (version 2019.4) [52]. The assessment of system stability was conducted using the root-mean-square deviation (RMSD) [53]. The molecular simulations were performed on a Lenovo P920 computer (Lenovo, Beijing, China), while the visualization of the data was carried out by using DSV.

### 4.3. Synthesis of Peptides

The peptides were manually synthesized on a Wang resin using the conventional Fmoc solid-phase synthesis approach [54]. The Fmoc group was removed using DMF solution containing 20% (*v*/*v*) piperidine for a maximum of 30 min. This was followed by three washes in isopropanol and DMF at room temperature. The Fmoc-amino acids were sequentially coupled to the peptide chain on the resin using a reaction mixture consisting of TBTU (0.91 g), DMF (10 mL), HOBt (0.45 g), and DIEA (0.52 mL) at room temperature for 2 h. The peptide chain was cleaved off the resin after the addition of the last amino acid. This cleavage process was performed in a combination of TFA, TIS, H_2_O, and EDT (94:1:2.5:2.5, *v*/*v*) at a temperature of 0 °C in ice water, with stirring at a speed of 3000 rpm. The crude peptides were purified using high-performance liquid chromatography (HPLC, LC-8A, SHIMADZU, Kyoto, Japan) on a reverse-phase C18 column to achieve a purity level of at least 95%. The isolated peptides were identified using a mass spectrometer (Agilent-6125B, Santa Clara, CA, USA).

### 4.4. Cytotoxicity of K5

Vero E6 and A549 cells were cultured in DMEM containing 10% FBS and 5% CO_2_ at 37 degrees centigrade (°C). The medium was renewed once every three days. The cells were passaged when they attained confluence.

The cytotoxicity of the functional peptide was assessed using the MTT test [55,56]. The test cells were seeded onto 96-well plates and grown overnight. The functional peptide K5 was introduced at concentrations of 0.1, 1, 10, and 100 μM for a 24 h incubation. MTT was then added, and cells were incubated for an additional 2 h. The medium was removed and replaced with 100 μL of DMSO. A measurement of absorbance was conducted at 492 nm (OD492) using a microplate reader (EnSpire, Cleveland, OH, USA). Cells without treatment were set as blanks, and the results are expressed as a percentage of the untreated cells.

### 4.5. Inhibition of SARS-CoV-2 S1 Protein Adsorption by K5

The cellular adsorption behaviors of K5 were examined using FITC-labeled peptide. Vero E6 cells were incubated in 24-well plates in medium containing 100 μM of FITC-labeled peptide for two different durations: 1 min and 1 h. Subsequently, the cells were washed three times using a PBS solution containing heparin, then stained with DAPI for 30 min. Finally, they were fixed using a 4% PFA solution and then observed using an inverted fluorescence microscope manufactured by Zeiss in Oberkochen, Germany [57]. The control group consisted of cells with only functional peptides added or only FITC added.

### 4.6. Inhibition of ACE2–S Protein Interaction by K5

To verify the ability of K5 to hinder the binding of ACE2 and the S protein, Vero E6 cells and A549 cells were cultured in 24-well plates for 24 h, then pre-incubated with K5 at different concentrations (ranging from 0.1 to 100 μM) for 1 h and washed with PBS containing heparin three times. Then, the Vero E6 cells were co-incubated in a 6 h treatment with 10 μg/mL SARS-CoV-2 S1 protein and rinsed three times. Following this, the SARS-CoV-2 S1 protein was stained with a primary antibody (anti-spike rabbit polyclonal primary antibody, 1:100) and an Alexa Fluor-labeled secondary antibody (goat anti-rabbit secondary antibody, 1:500) for 2 h and washed three times. Then, the cells were stained with DAPI for 30 min. Finally, the cells were washed three times with a PBS solution containing heparin and fixed with 4% PFA. The cells were sequentially treated with S1 protein, primary antibody, and secondary antibody under the same conditions. The control-group cells were not treated with K5 but were treated with S1 protein, primary antibody, and secondary antibody in the prescribed protocol under the same conditions.

The inhibitory capacity of K5 regarding ACE2–S protein interaction was evaluated by using a flow cytometer [58,59,60]. Cells were seeded at 1 × 10^5^ cells per well and cultivated in a 24-well plate. The cells underwent trypsinization, followed by centrifugation, and then were transferred to 1.5 mL tubes. For the sample group, the cells were pre-incubated with functional peptide at concentrations of 0.1, 1, 10, and 100 μM for 1 h, in PBS or FBS conditions. After centrifugation and washing three times, the cells were incubated with 10 μg/mL SARS-CoV-2 S1 protein for 1 h, in PBS or FBS conditions. Subsequently, the SARS-CoV-2 S1 protein was stained with a primary antibody (anti-spike rabbit polyclonal primary antibody, 1:100) and an Alexa Fluor-labeled secondary antibody (goat anti-rabbit secondary antibody, 1:500) for 2 h. Finally, the cells were centrifuged and washed three times. Fluorescence-activated cell sorting (FACS) analysis and a determination of the mean fluorescence intensity (MFI) were performed using a flow cytometer (Cytomics FC 500 MPL, Brea, CA, USA). Cells treated with only the primary and secondary antibodies were used as the blank group. The control-group cells were not treated with K5 but were treated with S1 protein, primary antibody, and secondary antibody in the prescribed protocol under the same conditions.

### 4.7. Biophysical Studies of K5

Circular dichroism (CD) spectroscopy was used to determine the secondary structures of K5. The CD spectra were scanned at 200 nm/min with a response time of 1 s and recorded in the range of 190–700 nm (Jasco-J810, Tokyo, Japan). The secondary structure of K5 was evaluated by Jasco-J810 analysis software (version 2.15.00).

To further determine the dynamic characteristics of K5, the ACE2 protein was coupled to activated Sepharose resin and shaken in an orbital shaker (Allsheng, Hangzhou, China) for 3 h. The supernatant was then extracted, and the residual protein content was determined using a micro-BCA assay (Thermo Fisher, Waltham, MA, USA) by measuring the UV absorbance at 280 nm. Then, a 1-M Gly solution was added for an additional 3 h of agitation to deactivate the residual coupling sites, followed by centrifugation and washing. Subsequently, functional peptide K5 solutions with varying concentrations, ranging from 0.1 to 1 mg/mL, were introduced into an orbital shaker for 3 h. Following this, the supernatant was removed and analyzed using a micro-BCA assay. The amount of bound peptide was calculated by a mass balance. The data were fitted to a Langmuir isotherm model [61]:

q = (q_m_ * C)/(K_d_ + C) (1)

where q, C, K_d_, and q_m_ are the concentration of bound protein (mg peptide/g resin), the concentration of free protein (mg peptide/mL solution), the dissociation constant (mg/mL), and the maximum capacity (mg peptide/g resin), respectively.

### 4.8. Statistical Analysis

The statistical significance of the data was evaluated using one-way analysis of variance (ANOVA). A *p*-value below 0.05 (*) was considered indicative of a statistically significant difference, while a *p*-value below 0.01 (**) was considered to indicate a high level of statistical significance. 

## 5. Conclusions

In this study, a novel functional peptide, KYPAY (K5), was developed to inhibit ACE2–S protein interaction through binding to the ACE2 LBD. Molecular simulations revealed that K5 has high affinity toward the ACE2 LBD. The functional peptide showed a significant cellular adsorption capacity, even after 1 min of incubation. Flow cytometry analysis demonstrated that the inhibition rate was 78% in the presence of 100 μM of peptide. Our study provides a promising candidate peptide drug for both preventing and treating COVID-19. Additionally, it strengthens the theoretical basis for developing new SARS-CoV-2 antagonists.

## Data Availability

All the data are included in the manuscript and additional file. All the materials and reagent sources used in this study are described in the methods section.

## Supplementary Materials

The following supporting information can be downloaded at: https://www.mdpi.com/article/10.3390/molecules29133022/s1, Figure S1: Topographic analysis of ACE2 LBD. Table S1: Docking results for candidate peptides binding to ACE2 LBD. Figure S2: The chemical formula of K5. Figure S3: The HPLC analysis of K5. Figure S4: The mass spectrometer analysis of K5. Figure S5: The cellular adsorption of K5 by A549 cells. Figure S6: The flow cytometry analysis of inhibitory effect of K5 on S protein–ACE2 interaction. Figure S7: Circular dichroic detection K5. Figure S8: Adsorption kinetics analysis of K5.

## Abbreviations

SARS-CoV-2	severe acute respiratory syndrome coronavirus 2
ACE2	angiotensin-converting enzyme-2
S1 protein	viral spike glycoprotein S1 subunit
S2 protein	viral spike glycoprotein S2 subunit
K5	KYPAY
RBD	receptor-binding domain
LBD	ligand-binding domain
FITC	fluorescein isothiocyanate
DMEM	Dulbecco’s modified Eagle’s medium
FBS	fetal bovine serum
PBS	phosphate buffer solution
DMSO	dimethyl sulfoxide
MTT	5-diphenyl-2-*H*-tetrazolium bromide
PFA	paraformaldehyde
DAPI	4′,6-diamidino-2-phenylindole
PDB	Protein Data Bank
MM-PBSA	Poisson–Boltzmann method
DSV	Discovery Studio Visualizer 4.5
MD	molecular dynamic
US	umbrella sampling
SPCE	extended simple point charge
RMSD	root-mean-square deviation
°C	degrees Celsius
OD492	opticaldensity492
min	minutes
h	hours
FACS	fluorescence-activated cell sorting
MFI	mean fluorescence intensity
ANOVA	one-way analysis of variance
E_binding_	binding free energy
ΔG	binding potential
Y5	YRLFR
CD	circular dichroism
