# Supplementary material for: Inhibition of ACE2–S Protein Interaction by a Short Functional Peptide with a Boomerang Structure
Source: Molecules. 2024 Jun 26;29(13):3022. doi: 10.3390/molecules29133022 (PMC11242946; doi:10.3390/molecules29133022)
Supplement: Supplementary file 1 [file molecules-29-03022-s001.zip › molecules-3037367-supplementary.pdf]

Article

Inhibition of ACE2–S Protein Interaction by a Short Functional Peptide with a Boomerang Structure

Yuping Wei [1,2,3], Ziyang Liu [1,2], Man Zhang [2,4], Xingyan Zhu [1,2], Qiuhong Niu [1,2] *

[1] School of Life Science, Nanyang Normal University, Nanyang 473061, China; fuziannwei@163.com (Y.W.); 18538992007@163.com (Z.L.); 18313948872@163.com (X.Z.)

[2] Research Center of Henan Provincial Agricultural Biomass Resource Engineering and Technology, Nanyang Normal University, Nanyang 473061, China; 18737779319@163.com

[3] State Key Laboratory of Biochemical Engineering, Institute of Process Engineering, Chinese Academy of Sciences, Beijing 100190, China

[4] Department of Oncology, Nanyang First People's Hospital, Nanyang 473002, China

* Correspondence: qiuhongniu723@163.com

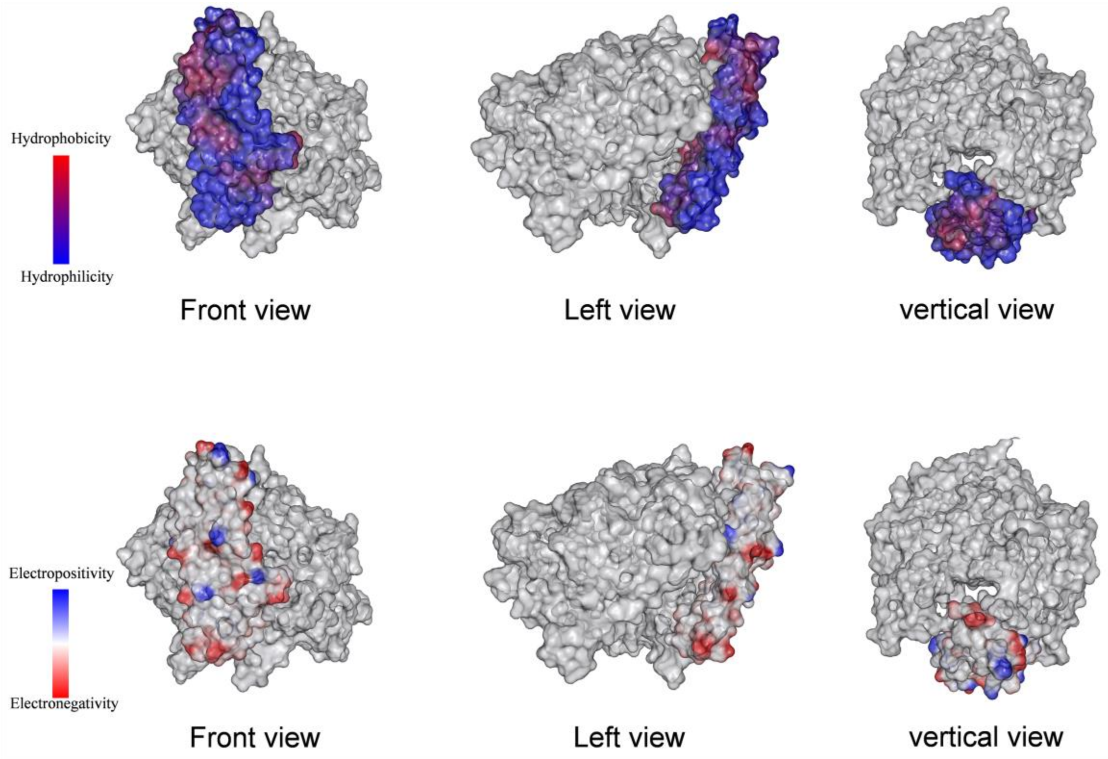

Figure S1. Topographic analysis of ACE2 LBD.

Table S1. Docking results for candidate peptides binding to ACE2 LBD.

	Sequence	$E_{Binding}$ energy (Kcal/mol)
**Control sequence**	YRLFR	-3.21
**Peptide**	RCPNG	-5.01
	RYPFL	-5.11
	KEPEQ	-5.22
	REPEQ	-5.32
	KRPEL	-5.36
	RLPAG	-5.50
	RLPRK	-5.56
	KYPPY	-5.58
	RYPWK	-5.59
	RGPAK	-5.60
	KYPFY	-5.62
	KAPWK	-5.67
	KYPQY	-5.75
	KYPMY	-5.93
	KYPAY	-6.91

The chemical formula of K5

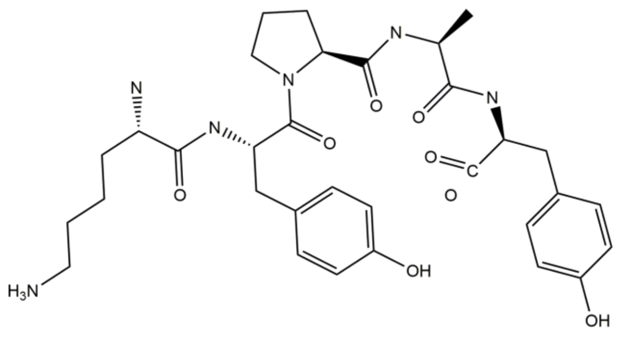

Figure S2. The chemical formula of K5.

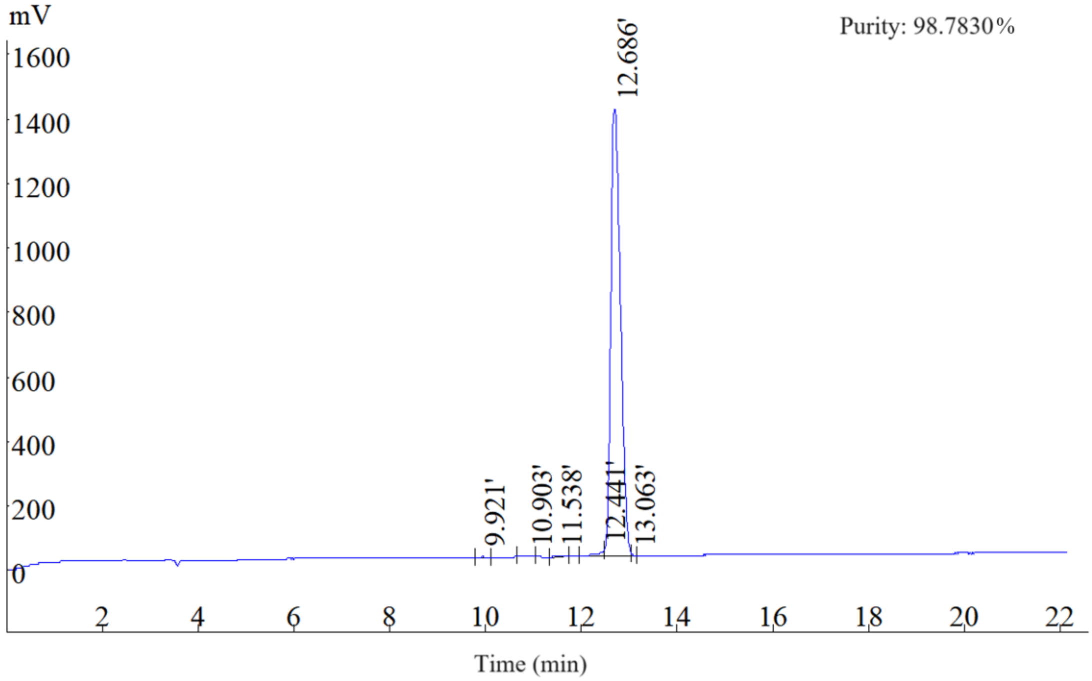

Figure S3. The HPLC analysis of K5. Elution gradient: from 11% acetonitrile to 36% acetonitrile

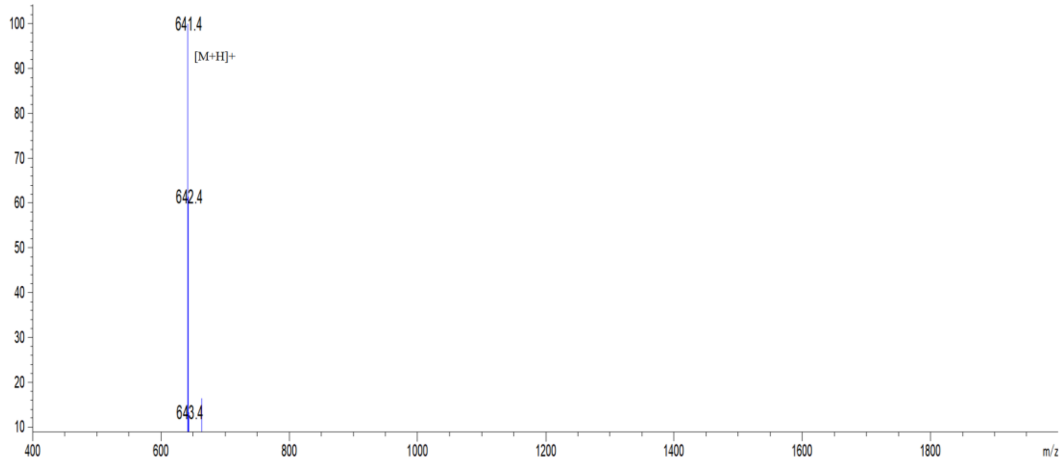

Figure S4. The mass spectrometer analysis of K5.

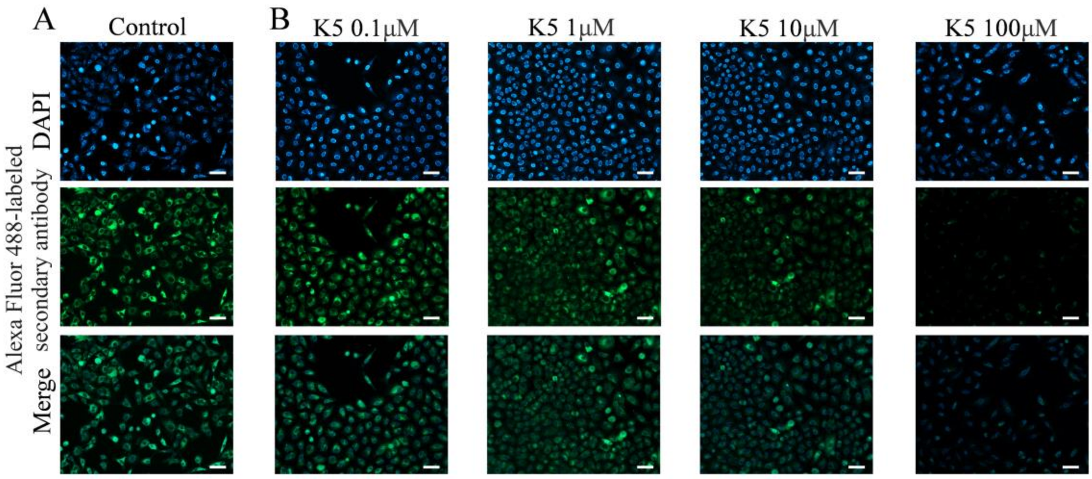

Figure S5. The cellular adsorption of K5 by A549 cells. (A) The fluorescence microscope images of control group for 1 hour and their distribution. (B) Visualization of SARS-CoV-2 S protein-ACE2 attachment blocking by K5. Bar = 50 μm.

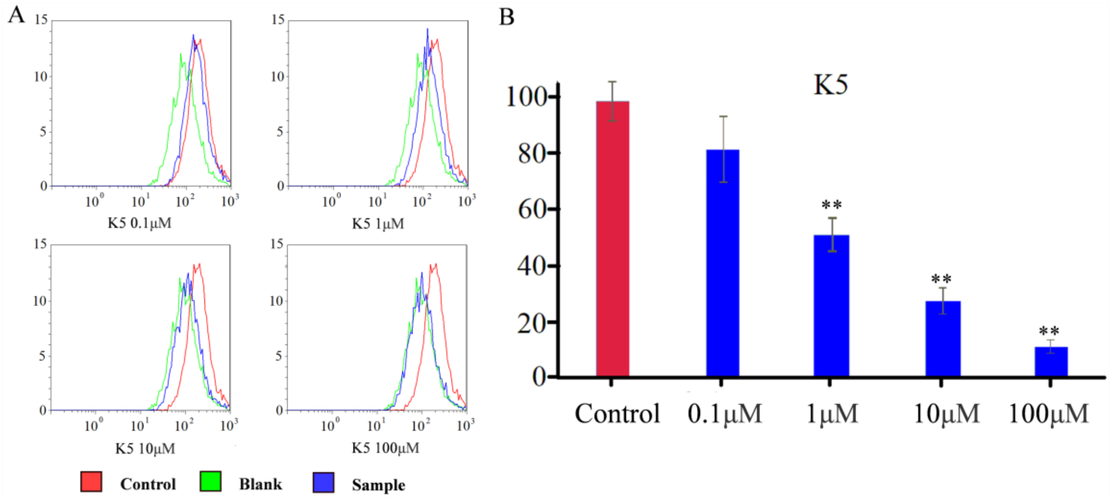

Figure S6. The flow cytometry analysis of inhibitory effect of K5 on S protein-ACE2 interaction. (A) FACS analysis of effect of K5 on SARS-CoV-2 S protein-A549 cell attachment. (B) MFI analysis of effect of K5 on SARS-CoV-2 S protein-A549 cell attachment.

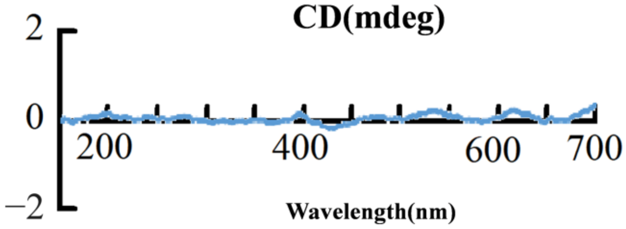

Figure S7. Circular dichroic analysis of K5.

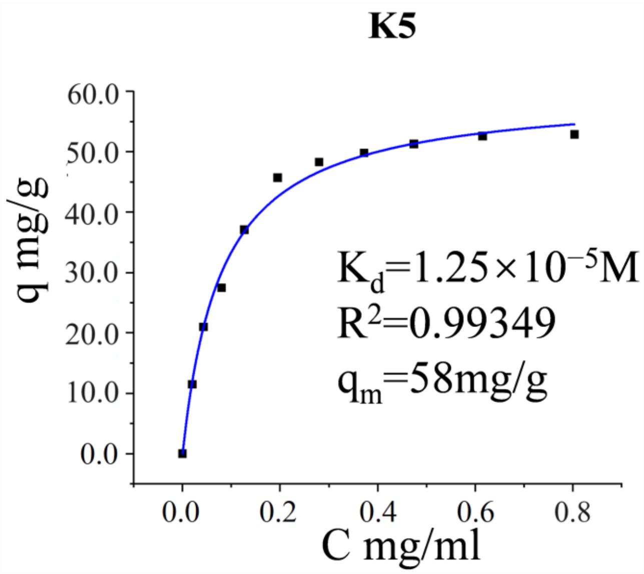

Figure S8. Adsorption kinetics analysis of K5.
